# PAI-1 Expression Is Required for HDACi-Induced Proliferative Arrest in *ras*-Transformed Renal Epithelial Cells

**DOI:** 10.1155/2011/710974

**Published:** 2011-09-06

**Authors:** Stephen P. Higgins, Craig E. Higgins, Paul J. Higgins

**Affiliations:** Center for Cell Biology and Cancer Research, Albany Medical College (MC-165), Albany, NY 12208, USA

## Abstract

Malignant transformation of mammalian cells with *ras* family oncogenes results in dramatic changes in cellular architecture and growth traits. The generation of flat revertants of v-K-*ras*-transformed renal cells by exposure to the histone deacetylase inhibitor sodium butyrate (NaB) was previously found to be dependent on transcriptional activation of the PAI-1 (SERPINE1) gene (encoding the type-1 inhibitor of urokinase and tissue-type plasminogen activators). NaB-initiated PAI-1 expression preceded induced cell spreading and entry into G_1_ arrest. To assess the relevance of PAI-1 induction to growth arrest in this cell system more critically, two complementary approaches were used. The addition of a stable, long half-life, recombinant PAI-1 mutant to PAI-1-deficient v-K-*ras*-/c-Ha-*ras*-transformants or to PAI-1 functionally null, NaB-resistant, 4HH cells (engineered by antisense knockdown of PAI-1 mRNA transcripts) resulted in marked cytostasis in the absence of NaB. The transfection of *ras*-transformed cells with the Rc/CMVPAI expression construct, moreover, significantly elevated constitutive PAI-1 synthesis (10- to 20-fold) with a concomitant reduction in proliferative rate. These data suggest that high-level PAI-1 expression suppresses growth of chronic *ras*-oncogene transformed cells and is likely a major cytostatic effector of NaB exposure.

## 1. Introduction

Histone acetyltransferases (HATs) transfer acetyl groups from acetyl CoA to specific lysine residues in the amino terminal histone “tails” to form *ε*-N-acetyl lysine promoting an “open” or relaxed chromatin structure. Several transcriptional coactivators, including CBP/p300 and SRC, have intrinsic HAT activity [1, 2]. Histone deacetylases (HDACs), in contrast, catalyze the removal of acetyl groups on target lysines [3, 4] creating a condensed, transcriptionally repressed, chromatin organization [5]. Of the various HDAC inhibitors (HDACi), several exhibit more or less specificity for individual members of the four classes (I-VI) of human HDACs [6, 7]. 

 A major mode of action of HDACi (i.e., the transcription-dependent mechanism) [5] affects gene reprogramming as a consequence of HDACi type, concentration, and duration of exposure [8, 9]. Recent estimates place the number of HDACi-impacted genes at 2–10% of the total expressed repertoire, several of which negatively regulate cell cycle progression [10–13] such as p21^WAF1/CIP1^ and plasminogen activator inhibitor type-1 (PAI-1; SERPINE1) [14–21]. PAI-1 is particularly relevant in this context as this SERPIN complexes with both urokinase (uPA) and tissue-type (tPA) plasminogen activators to limit pericellular plasmin generation effectively attenuating uPA-/plasmin-dependent growth factor activation and cellular proliferative responses [22, 23]. PAI-1, in fact, is both necessary and sufficient for p53-dependent growth arrest [23–26] and required for TGF-*β*1-mediated antiproliferative effects in human keratinocytes and mouse embryo fibroblasts [27]. Activated *ras *or *raf* oncogenes trigger the initiation of a senescence-like growth arrest program, with induction of PAI-1, in several cell types [28–31]. At least some cells transformed as a consequence of chronic oncogenic *ras* expression, and that escape *ras*-induced senescence can also undergo proliferative arrest upon exposure to certain HDACi (e.g., sodium butyrate; NaB) with concomitant high-level PAI-1 induction [16, 17, 32]. It is not known, however, if HDACi-associated growth inhibition of immortalized *ras*-transformants, like entrance of normal cells into replicative senescence [23], also requires PAI-1-induction. 

 This paper details the involvement of PAI-1 expression during HDACi-induced growth restriction in several well-characterized v-Ki-*ras* and Ha-*ras* ^val-12^-transformed epithelial cell lines [15, 16]. NaB was selected for this study as this HDACi is a potent stimulator of PAI-1 expression [14, 20] and cytostasis [16] in *ras*-transformed renal cells. NaB-mediated growth inhibition was not evident in PAI-1 knockdown (i.e., anti-sense) cells, but the HDACi-dependent proliferative block could be rescued by vector-driven PAI-1 overexpression.

## 2. Materials and Methods

### 2.1. Culture Conditions and Engineered Cells

The various *ras*-transformed renal epithelial cell lines used in this study [15, 16] grow in serum-free DMEM (at least over the short time frame used in this study; 3–5 days) facilitating assessments of the proliferation-modulating effects of NaB (1–10 mM) and exogenous PAI-1 (0.02–100 nM stable mutant 14-1B, *t*_1/2_ = 145 hours; N150H, K154T, Q319L, M354I) [33] in both the presence and absence of FBS. The derivation of the PAI-1 functionally null knockdown (PAI-1^KD^) 4HH cell line by transfection of a 2.6 kb rat PAI-1 *Eco*R1/*Hin*dIII cDNA fragment (representing nucleotides −118 to +2572) cloned in anti-sense orientation (Rc/CMVIAP) has been described [34, 35]. v-*ras*-transformed cells were also transfected with the Rc/CMVPAI sense vector to initiate high-level PAI-1 expression in the absence of NaB or with the empty Rc/CMV construct [32]. Coupled *in vitro* transcription/translation assay confirmed that a full-length immunoreactive PAI-1 protein was synthesized using the Rc/CMVPAI vector as a template [35]. In some cases, Rc/CMVPAI transfectants were selected with G418 [32]. Cloning strategy and cell line derivation are detailed in the text. c-Ha-*ras* oncogene-expressing human HaCaT II-4 keratinocytes were described previously [33, 36] as were the PAI-1-deficient and reconstituted renal cell lines [35].

### 2.2. Northern Blotting

Cytoplasmic RNA was separated by electrophoresis on denaturing 1% agarose/2.2 M formaldehyde gels, transferred to nitrocellulose and blots hybridized with a ^32^P-labeled EcoRI-HindIII fragment of rat PAI-1 cDNA (specific activity 1-2 × 10^8^ cpm/*μ*g DNA) for 48 hr at 4°C. The recombinant pBluescript (SK(-) phagemid pRPAISS1-3, containing a 3.0-kb EcoRI/SstII-flanked cDNA insert encoding PAI-1, was used for isolation of the pRPAImr1-4 probe used for hybridization. Briefly, pRPAISS1-3 was digested with EcoRI/Hind III at 37°C for 1 hr and fragments separated in 1% agarose gels. After staining with ethidium bromide, bands representing the PAI-1 cDNA insert were excised and electroeluted. This insert fragment (pRPAImr1-4) was labeled with ^32^P-dCTP by random priming. Following hybridization, membranes were washed sequentially for 20 minutes each in 2x SSC/0.1% SDS (twice) and then in 1x SSC/0.1% SDS, all at 55°C.

### 2.3. Extraction of Metabolically Labeled Cells and Gel Electrophoresis

Growth media (in 35-mm diameter cultures) were aspirated, cells washed twice with HBSS and 1 mL of labeling medium (FBS- and methionine-free RPMI 1640 medium containing 50 *μ*Ci ^35^S-methionine (specific activity = 1100 Ci/mmol) added to each culture. At the end of a 6 hr labeling period, the substrate adherent-enriched (SAP) cellular fraction was collected, clarified at 13,000 ×g, and solubilized in lysis buffer (9.8 M urea, 2% Nonidet P-40, 2% ampholytes, and 100 mm dithiothreitol) [37]. 1-D gel separations were as detailed previously [38]. For 2-D gel electrophoresis, 50,000 cpm ^35^S-methionine-labeled protein were loaded onto prerun 1.5 mm diameter tube gels (9.1 M urea, 2% Nonidet P-40, 6% pH 5–7 ampholytes, 1.2% pH 3–10 ampholytes, 4% acrylamide/bisacrylamide for isoelectric focusing (IEF) for 18 hr prior to separation on SDS-10% acrylamide slab gels [39]. Individual protein spots were mapped and quantitated with a Bio-Image Investigator 2-D Electrophoresis Analysis system interfaced to a SUN SPARC workstation [40].

## 3. Results

### 3.1. PAI-1 Induction in v-*ras*-Transformed Renal Cells upon Exposure to the HDACi NaB

**Figure 1:**
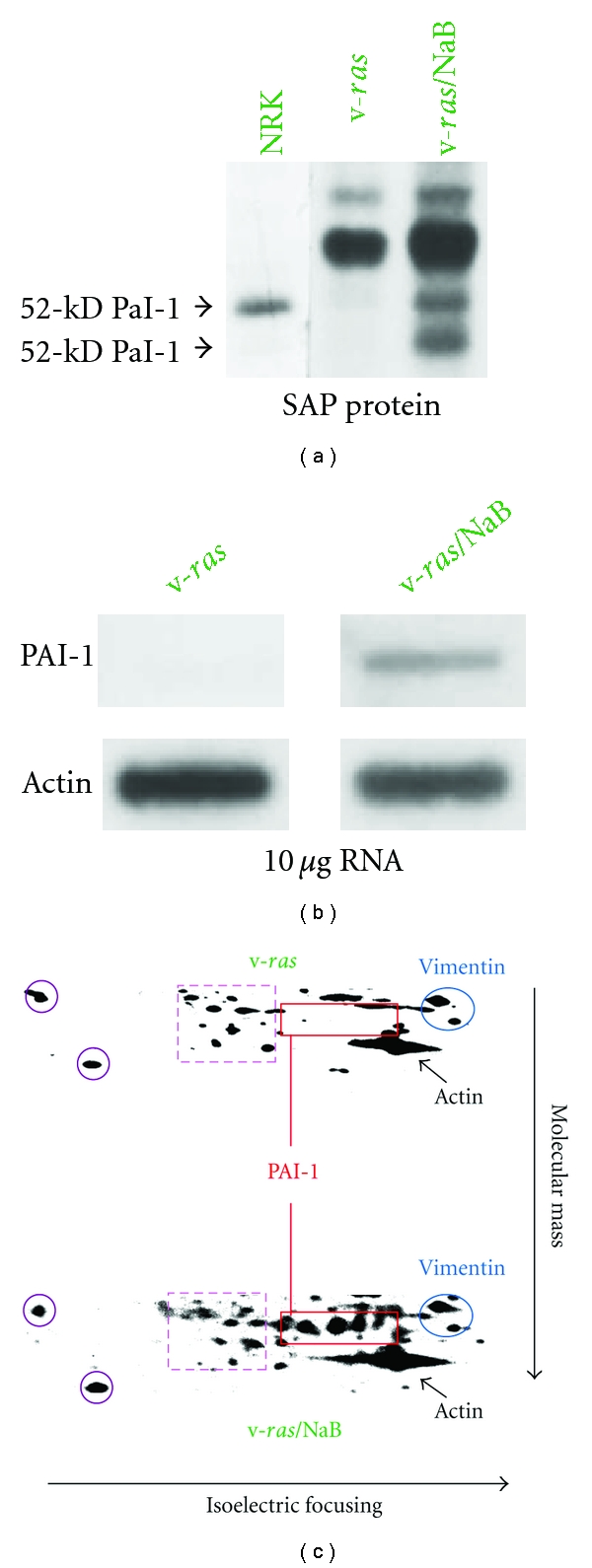
Electrophoresis of the ^35^S-methionine-labeled saponin-resistant (SAP) protein fraction of v-*ras*-transformed renal cells and their NaB-treated counterparts indicated that PAI-1 (both the 50-kD and fully glycosylated 52-kD species) were expressed in NaB revertants but not in untreated cells (a). PAI-1 from normal kidney cells (NRK) served as a marker. The band above PAI-1 in v-*ras* cells (at 62-kD) is the heavily-glycosylated forms of osteopontin (a). Northern blotting confirmed the absence of PAI-1 mRNA in v-*ras*-transformants and the restoration of mRNA expression in response to NaB (b). 2-D electrophoretic mapping of the SAP fraction proteins derived from ^35^S-methionine-labeled cultures revealed, furthermore, that PAI-1 induction in response to NaB treatment was rather selective (c). Map positions of the glycosylated PAI-1 isoforms are indicated (solid red outlined rectangle). Proteins common between the cell types are highlighted in color (purple circles, red dashed line box, blue ovals indicating vimentin, and phosho-vimentin breakdown products and actin by black arrows) and did not change in abundance despite the significant PAI-1 induction evident in the v-*ras*/NaB protein profile (c).

**Figure 2:**
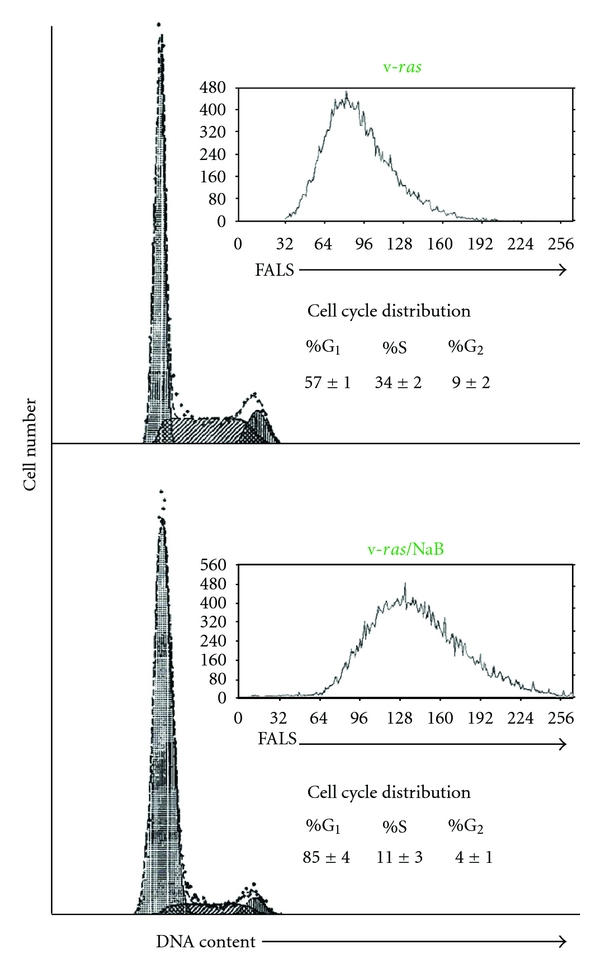
Impact of NaB on cell cycle progression in v-*ras* transformants. The S-phase fraction of exponentially growing (10% FBS) cells approximated >34% with a tight mean cellular size distribution (mean: channel 80) as assessed by forward angle light scatter (FALS) measurements. NaB treatment resulted in a G_1_ block (even in FBS-supplemented medium) and a significantly increased mean size.

**Figure 3:**
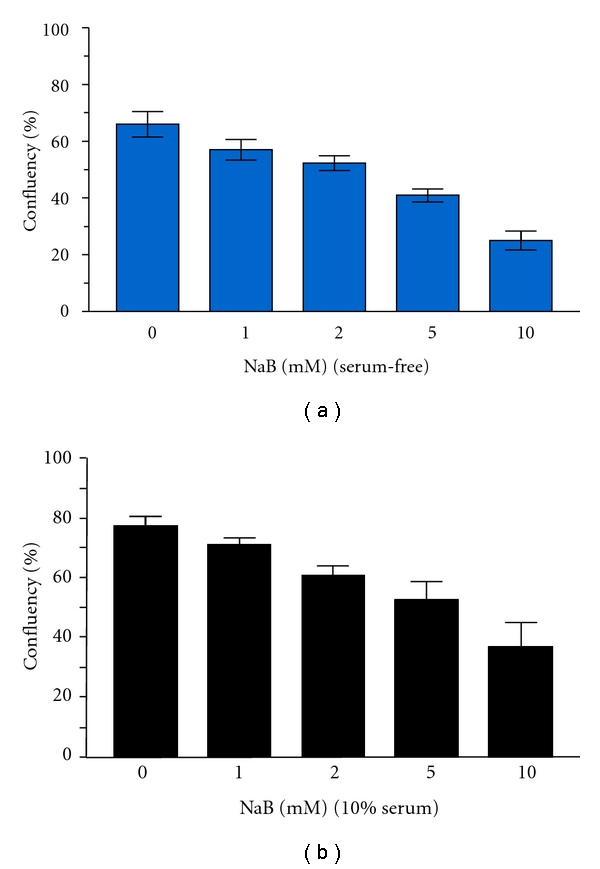
NaB suppressed cell growth in serum-free or supplemented culture conditions. Proliferative restriction was maximal at 10 mM resulting in final population densities of just 47% (a) and 52% (b) compared to respective controls. Data plotted is the mean ± standard deviation for triplicate assessments of final cell densities (i.e., % confluency) for each NaB concentration under the two growth conditions.

**Figure 4:**
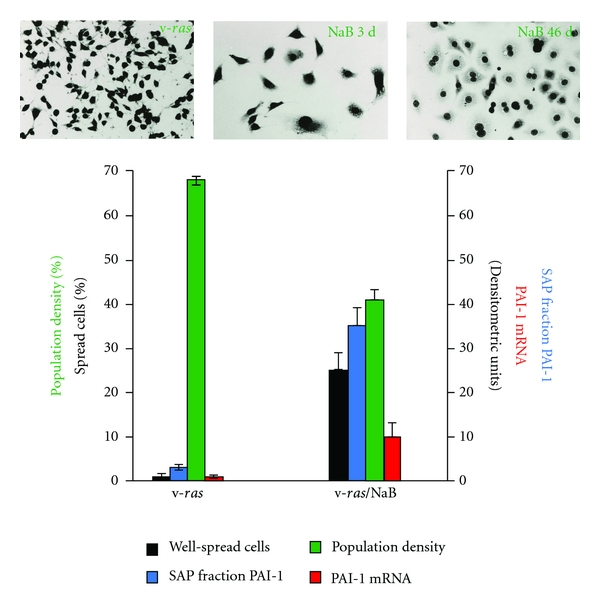
Summary characteristics of the NaB-induced phenotype in v-*ras*-transformed renal epithelial cells. NaB initiated a rapid cytostasis evident within 3 days after exposure with the acquisition of increased cell-spread area consistent with FALS assessment of cell size (i.e., Figure 2). Cells remained growth arrested even after protracted treatment (e.g., 46 days) although such long-term cultures had an increased binucleate frequency. PAI-1 mRNA/protein expression in control populations was low to undetectable in contrast to the levels of PAI-1 transcripts and protein evident in response to NaB. Plots illustrate the mean ± standard deviation for triplicate assessments for each of the parameters evaluated.

Limited expression profiling previously indicated that PAI-1 was among the most abundant of the NaB-upregulated genes in *ras*-transformed renal epithelial cells [14, 15] consistent with microarray and bioinformatic analyses of genetic networks responsive to NaB in colonic epithelial cells [20]. 1-D electrophoresis (Figure 1(a)), northern blotting (Figure 1(b)), and 2-D proteomic mapping (Figure 1(c)), moreover, confirmed a significant and rather selective PAI-1 induction in NaB-stimulated v-*ras*-transformants (involving both the 50-kD and mature 52-kD glycosylated PAI-1 species), using 1-D/2-D mobility and immunochemical identification criteria established previously [16, 38, 41], relative to nondetectable PAI-1 levels in control v-*ras* populations. PAI-1 upregulation correlated with a prominent NaB-associated G_1_ arrest increased cell size (Figure 2) and concentration-dependent proliferative inhibition resulting in a 63% (Figure 3(a)) and 48% (Figure 3(b)) decrease in population density in serum-free and 10% serum-supplemented medium, respectively (summarized in Figure 4). Collectively, these findings (Figures 1 and 4) are consistent with the conclusion that v-*ras*-transformants that escape *ras* oncogene-initiated cellular senescence [29, 42, 43] are essentially PAI-1 null.

### 3.2. Growth Arrest in *ras*-Transformants Is Restored by Exogenous Exposure to a Long Half-Life PAI-1 Mutant or by Vector-Driven Reconstitution of PAI-1 Expression

**Figure 5:**
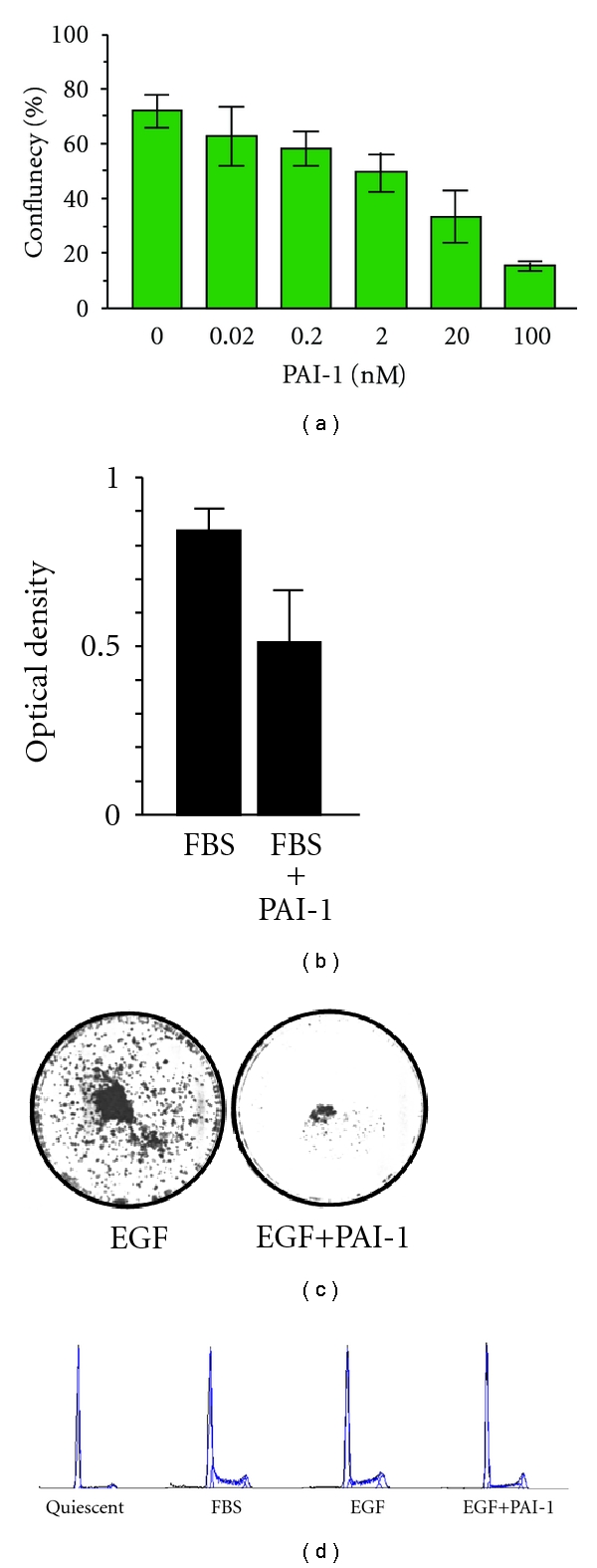
Increasing concentrations of PAI-1 (range 0.02 to 100 nM) effectively suppressed growth of v-*ras*-transformants in serum-free (a) as well as serum-supplemented (b) media. In (b), PAI-1 was added to a final level of 20 nM. PAI-1 also inhibited EGF-induced HaCaT proliferation, as assessed by crystal violet staining of cells stimulated with EGF (10 ng/mL) for 5 days in the absence or presence of PAI-1 (20 nM) (c). Growth arrest in EGF-treated HaCaT keratinocytes reflected a PAI-1-induced G_1_ block evident as early as 24 hours after the addition of PAI-1 compared to the prominent S-phase cohort in FBS- or control EGF-treated cultures (d). Starting quiescent populations (1 day in serum-free medium) were virtually devoid of DNA-synthesizing cells.

**Figure 6:**
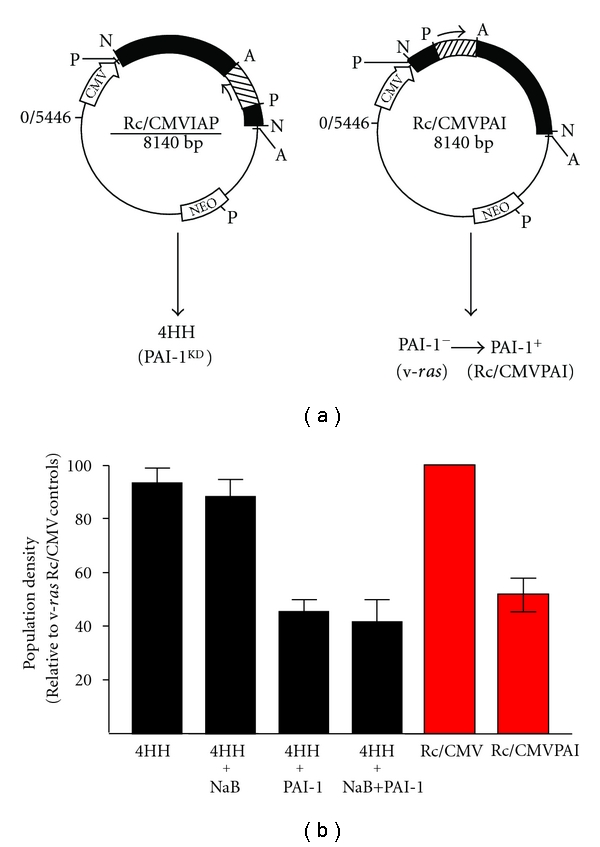
PAI-1 antisense and sense expression vectors were used to generate the PAI-1 knockdown (4HH; PAI-1^KD^) and overexpressing (Rc/CMVPAI) cell lines, respectively, (a). PAI-1-deficient 4HH cells were resistant to NaB-mediated cytostasis but remained sensitive to PAI-1-induced growth arrest. The combination of NaB+PAI-1 reduced final population densities similar to that of PAI-1 alone cultures (b). Vector-driven PAI-1 overexpression in v-*ras* transformants also inhibited cell growth consistent with results of the PAI-1 add-back experiments (e.g., Figure 5). Data plotted represents the mean ± standard deviation for triplicate assessments of final cell densities (i.e., % confluency).

Since targeted suppression of PAI-1 leads to bypass of both replicative senescence and TGF-*β*-induced growth arrest [23, 27], it was important to determine if exogenously-delivered PAI-1 could similarly regulate the proliferative response of PAI-1-deficient *ras* transformants in the absence of NaB. The addition of a long half-life recombinant PAI-1 mutant (PAI-1 14-1B) effectively suppressed growth of v-*ras*-transformed cells in a concentration-dependent manner with an 80% reduction in final population density after a 5-day exposure to 100 nM PAI-1 (Figure 5(a)). Indeed, the level of growth inhibition in cultures exposed to 20 nM PAI-1 (45% reduction in population density relative to the corresponding control) (Figure 5(a)) approximated the 47.5% decrease induced by 10 mM NaB even in the presence of serum (Figure 4(b)). Ha-*ras*-transformed HaCaT cells, which express low levels of PAI-1 in response to EGF [33], were also growth inhibited by exposure to PAI-1 14-1B in the presence of FBS or EGF (Figures 5(b) and 5(c)) largely due to G_1_ arrest (Figure 5(c)). To assess this effect more critically in a genetic context, antisense knockdown (PAI-1^KD^; 4HH) cells (Figure 6(a)), which are resistant to NaB-dependent proliferative inhibition, were incubated in PAI-1-supplemented medium with or without, addition of NaB. Recombinant PAI-1, at a final concentration of 20 nM, effectively suppressed PAI-1^KD^ cell proliferation; the combination of PAI-1 + NaB did not significantly impact the extent of cytostasis compared to PAI-1 alone (Figure 6(b)). Transient vector-driven re-expression of PAI-1 in Rc/CMVPAI v-*ras *transfectants (Figure 6(a)) similarly reduced cell growth relative to cells transfected with the empty Rc/CMV construct (Figure 6(b)). Mass cultures of Rc/CMVPAI-expressing cells and, in particular, their G418-selected clonal isolates, but not cells transfected with Rc/CMV without the 2.6 kb PAI-1 cDNA insert, had significant numbers of very well-spread cells (a hallmark of the growth arrest phenotype in renal epithelial cells [14–16]) compared to Rc/CMV populations. The marked reduction in cell proliferation (Figure 6(b)) and increased spreading in Rc/CMVPAI as compared to Rc/CMV transfectants correlated with an approximately 22-fold increase in PAI-1 expression.

## 4. Discussion

Data mining of microarray and serial analysis of gene expression profiles consistently identified increased PAI-1 levels as characteristic of specific growth arrest states (e.g., [23, 27, 35, 36, 39, 42–46]). Similar to other HDACi-regulated genes, several of which negatively regulate cell cycle progression [10–13], PAI-1 is a particularly relevant candidate as this SERPIN attenuates uPA-/plasmin-dependent growth factor activation and cellular proliferative responses [22, 23], mediates p53-dependent cytostasis [23–26], and is required for TGF-*β*1-mediated antiproliferative responses [27]. In cells expressing activated *ras *or *raf* oncogenes, moreover, induced PAI-1 initiates the engagement of a senescence-like phenotype [28–31] while, for those cells that escape *ras*-induced senescence, the growth arrest program can be “rescued” upon exposure to certain HDACi (e.g., NaB) with concomitant high-level PAI-1 induction [16, 17, 32]. While molecular events underlying NaB-stimulated PAI-1 expression is unclear, NaB enhances Smad3 phosphorylation and potentiates TGF-*β*-induced PAI-1 expression [47], concomitant with NaB-induced G_1_ arrest [48]. Indeed, overexpression of SMAD3 in v-Ha-*ras*-transformed keratinocytes induced a cytostatic response, stimulated PAI-1 promoter (3TP-Lux reporter)-dependent transcription, and increased the incidence of senescent epithelial cells [49]. The present findings are consistent with these and previous data that TGF-*β*-initiated growth inhibition as well as senescence arrest is PAI-1-dependent [23, 27] and establish, moreover, PAI-1 as a mediator of NaB-initiated cytostasis. Whether this response can be adapted for directed “senescence therapy” of human cancers, remains to be assessed.

 NaB upregulates the cell cycle inhibitors p21^WAF1/CIP1^ and p16^INK4A^ in human fibroblasts although targeted disruption of p21 only weakly impacted HDACi-induced senescence-like growth arrest. p53^−/−^ mouse embryo fibroblasts (MEFs), moreover, are resistant to NaB-initiated cytostasis indicating that this tumor suppressor is a major senescence determinant in MEFs [50], and NaB-mediated apoptosis in human melanoma cells is p53-dependent [51]. Indeed, nutlin-3, an MDM2 inhibitor which restores p53 function in tumor cells that retain a wild-type p53, cooperate with several HDACis (including NaB) to induce cell death in p53 wild-type tumor cell lines but not in p53-null PC-3 prostatic carcinoma likely by HDACi-induced p53 hyperacetylation and/or MDM2/MDM4 downregulation [52]. This may be dependent, in part, on the extent of increased p53 expression in response to NaB [53]. Similarly, NaB-stimulated p53 transcriptional activity initiated irreversible G_1_/S cell cycle arrest in c-Ha-*ras*-transformed rat embryo fibroblasts that were p53 wild-type but not in cells with an inactivated p53 [54]. While the actual contribution of p21 versus INK4A/ARF-encoded genes (e.g., p19) in NaB-induced growth arrest is uncertain [55, 56], the role of p53 (at least in MEFs) may be more relevant since p53 is required for PAI-1 expression and growth arrest (see [27, 57]; and Overstreet et al., in preparation). p53 status, therefore, may be a major aspect of HDACi-induced cell cycle arrest through its transcriptional control of PAI-1 and, thereby, PAI-1-dependent cytostasis.

## References

[1] Marmorstein R, Roth SY (2001). Histone acetyltransfe*ras*es: function, structure, and catalysis. *Current Opinion in Genetics and Development*.

[2] Garcia-Manero G, Issa JP (2005). Histone deacetylase inhibitors: a review of their clinical status as antineoplastic agents. *Cancer Investigation*.

[3] Roth SY, Denu JM, Allis CD (2001). Histone acetyltransfe*ras*es. *Annual Review of Biochemistry*.

[4] Thiagalingam S, Cheng KH, Lee HJ, Mineva N, Thiagalingam A, Ponte JF (2003). Histone deacetylases: unique players in shaping the epigenetic histone code. *Annals of the New York Academy of Sciences*.

[5] Xu WS, Parmigiani RB, Marks PA (2007). Histone deacetylase inhibitors: molecular mechanisms of action. *Oncogene*.

[6] Davie JR (2003). Inhibition of histone deacetylase activity by butyrate. *Journal of Nutrition*.

[7] Bolden JE, Peart MJ, Johnstone RW (2006). Anticancer activities of histone deacetylase inhibitors. *Nature Reviews Drug Discovery*.

[8] Chambers AE, Banerjee S, Chaplin T (2003). Histone acetylation-mediated regulation of genes in leukaemic cells. *European Journal of Cancer*.

[9] Sasakawa Y, Naoe Y, Sogo N (2005). Marker genes to predict sensitivity to FK228, a histone deacetylase inhibitor. *Biochemical Pharmacology*.

[10] Ocker M, Schneider-Stock R (2007). Histone deacetylase inhibitors: signalling towards p21^cip1/waf1^. *International Journal of Biochemistry and Cell Biology*.

[11] van Lint C, Emiliani S, Verdin E (1996). The expression of a small fraction of cellular genes is changed in response to histone hyperacetylation. *Gene Expression*.

[12] Glaser KB, Staver MJ, Waring JF, Stender J, Ulrich RG, Davidsen SK (2003). Gene expression profiling of multiple histone deacetylase (HDAC) inhibitors: defining a common gene set produced by HDAC inhibition in T24 and MDA carcinoma cell lines. *Molecular Cancer Therapeutics*.

[13] Gartel AL, Radhakrishnan SK (2005). Lost in transcription: p21 repression, mechanisms, and consequences. *Cancer Research*.

[14] Ryan MP, Higgins PJ (1989). Sodium-n-butyrate induces secretion and substrate accumulation of p52 in Kirsten sarcoma virus-transformed rat kidney fibroblasts. *International Journal of Biochemistry*.

[15] Higgins PJ, Ryan MP (1991). P52(PAI-1) and actin expression in butyrate-induced flat revertants of v-*ras*-transformed rat kidney cells. *Biochemical Journal*.

[16] Higgins PJ, Chaudhari P, Ryan MP (1991). Cell-shape regulation and matrix protein p52 content in phenotypic variants of *ras*-transformed rat kidney fibroblasts. Functional analysis and biochemical comparison of p52 with proteins implicated in cell-shape determination. *Biochemical Journal*.

[17] Mariadason JM, Corner GA, Augenlicht LH (2000). Genetic reprogramming in pathways of colonic cell maturation induced by short chain fatty acids: comparison with trichostatin A, sulindac, and curcumin and implications for chemoprevention of colon cancer. *Cancer Research*.

[18] Chiba T, Yokosuka O, Arai M (2004). Identification of genes up-regulated by histone deacetylase inhibition with cDNA microarray and exploration of epigenetic alterations on hepatoma cells. *Journal of Hepatology*.

[19] Wakabayashi K, Saito H, Kaneko F, Nakamoto N, Tada S, Hibi T (2005). Gene expression associated with the decrease in malignant phenotype of human liver cancer cells following stimulation with a histone deacetylase inhibitor. *International Journal of Oncology*.

[20] Tabuchi Y, Takasaki I, Doi T, Ishii Y, Sakai H, Kondo T (2006). Genetic networks responsive to sodium butyrate in colonic epithelial cells. *FEBS Letters*.

[21] Ocker M, Schneider-Stock R (2007). Histone deacetylase inhibitors: signalling towards p21^cip1/waf1^. *International Journal of Biochemistry and Cell Biology*.

[22] Wilkins-Port CE, Ye Q, Mazurkiewicz JE, Higgins PJ (2009). TGF-*β*1 + EGF-initiated invasive potential in transformed human keratinocytes is coupled to a plasmin/mmp-10/mmp-1-dependent collagen remodeling axis: role for PAI-1. *Cancer Research*.

[23] Kortlever RM, Higgins PJ, Bernards R (2006). Plasminogen activator inhibitor-1 is a critical downstream target of p53 in the induction of replicative senescence. *Nature Cell Biology*.

[24] Klein LE, Freeze BS, Smith AB, Horwitz SB (2005). The microtubule stabilizing agent discodermolide is a potent inducer of accelerated cell senescence. *Cell Cycle*.

[25] Ota H, Tokunaga E, Chang K (2006). Sirt1 inhibitor, Sirtinol, induces senescence-like growth arrest with attenuated *ras*-MAPK signaling in human cancer cells. *Oncogene*.

[26] Schmitt CA, Fridman JS, Yang M (2002). A senescence program controlled by p53 and p16^INK4a^ contributes to the outcome of cancer therapy. *Cell*.

[27] Kortlever RM, Nijwening JH, Bernards R (2008). Transforming growth factor-*β* requires its target plasminogen activator inhibitor-1 for cytostatic activity. *Journal of Biological Chemistry*.

[28] Mason DX, Jackson TJ, Lin AW (2004). Molecular signature of oncogenic *ras*-induced senescence. *Oncogene*.

[29] Serrano M, Lin AW, McCurrach ME, Beach D, Lowe SW (1997). Oncogenic *ras* provokes premature cell senescence associated with accumulation of p53 and p16^^INK4a^^. *Cell*.

[30] Gorgoulis VG, Halazonetis TD (2010). Oncogene-induced senescence: the bright and dark side of the response. *Current Opinion in Cell Biology*.

[31] Gabai VL, Yaglom JA, Waldman T, Sherman MY (2009). Heat shock protein Hsp72 controls oncogene-induced senescence pathways in cancer cells. *Molecular and Cellular Biology*.

[32] Higgins PJ, Ryan MP, Jelley DM (1997). p52^PAI-1^ gene expression in butyrate-induced flat revertants of v-*ras*-transformed rat kidney cells: mechanism of induction and involvement in the morphological response. *Biochemical Journal*.

[33] Berkenpas MB, Lawrence DA, Ginsburg D (1995). Molecular evolution of plasminogen activator inhibitor-1 functional stability. *EMBO Journal*.

[34] Providence KM, Kutz SM, Staiano-Coico L, Higgins PJ (2000). PAI-1 gene expression is regionally induced in wounded epithelial cell monolayers and required for injury repair. *Journal of Cellular Physiology*.

[35] Higgins PJ, Ryan MP (1989). Biochemical localization of the transformation-sensitive 52 kDa p52 protein to the substratum contact regions of cultured rat fibroblasts. Butyrate induction, characterization, and quantification of p52 in v-*ras* transformed cells. *Biochemical Journal*.

[36] Ryan MP, Higgins PJ (1988). Cytoarchitecture of Kirsten sarcoma virus-transformed rat kidney fibroblasts: butyrate-induced reorganization within the actin microfilament network. *Journal of Cellular Physiology*.

[37] Higgins PJ, Smith TJ (1993). Pleotrophic action of interferon gamma in human orbital fibroblasts. *Biochimica et Biophysica Acta*.

[38] Higgins PJ, Ryan MP, Zehab R, Gelehrter TD, Chaudhari P (1990). p52 Induction by cytochalasin D in rat kidney fibroblasts: homologies between p52 and plasminogen activator inhibitor type-1. *Journal of Cellular Physiology*.

[39] Higgins PJ, Ryan MP, Ahmed A (1992). Cell-shape-associated transcriptional activation of the p52(PAI-1)gene in rat kidney cells. *Biochemical Journal*.

[40] Yaswen P, Campisi J (2007). Oncogene-induced senescence pathways weave an intricate tapestry. *Cell*.

[41] Kilbey A, Terry A, Cameron ER, Neil JC (2008). Oncogene-induced senescence: an essential role for Runx. *Cell Cycle*.

[42] Shelton DN, Chang E, Whittier PS, Choi D, Funk WD (1999). Microarray analysis of replicative senescence. *Current Biology*.

[43] Untergasser G, Koch HB, Menssen A, Hermeking H (2002). Characterization of epithelial senescence by serial analysis of gene expression: identification of genes potentially involved in prostate cancer. *Cancer Research*.

[44] Fridman AL, Tainsky MA (2008). Critical pathways in cellular senescence and immortalization revealed by gene expression profiling. *Oncogene*.

[45] Freytag J, Wilkins-Port CE, Higgins CE (2009). PAI-1 regulates the invasive phenotype in human cutaneous squamous cell carcinoma. *Journal of Oncology*.

[46] Freytag J, Wilkins-Port CE, Higgins CE, Higgins SP, Samarakoon R, Higgins PJ (2010). PAI-1 mediates the TGF-*β*1+EGF-induced "scatter" response in transformed human keratinocytes. *Journal of Investigative Dermatology*.

[47] Nguyen KA, Cao Y, Chen JR, Townsend CM, Ko TC (2006). Dietary fiber enhances a tumor suppressor signaling pathway in the gut. *Annals of Surgery*.

[48] Pajak B, Orzechowski A, Gajkowska B (2007). Molecular basis of sodium butyrate-dependent proapoptotic activity in cancer cells. *Advances in Medical Sciences*.

[49] Vijayachandra K, Lee J, Glick AB (2003). Smad3 regulates senescence and malignant conversion in a mouse multistage skin carcinogenesis model. *Cancer Research*.

[50] Munro J, Barr NI, Ireland H, Morrison V, Parkinson EK (2004). Histone deacetylase inhibitors induce a senescence-like state in human cells by a p16-dependent mechanism that is independent of a mitotic clock. *Experimental Cell Research*.

[51] Bandyopadhyay D, Mishra A, Medrano EE (2004). Overexpression of histone deacetylase 1 confers resistance to sodium butyrate-mediated apoptosis in melanoma cells through a p53-mediated pathway. *Cancer Research*.

[52] Palani CD, Beck JF, Sonnemann J Histone deacetylase inhibitors enhance the anticancer activity of nutlin-3 and induce p53 hyperacetylation and downregulation of MDM2 and MDM4 gene expression.

[53] Joseph J, Wajapeyee N, Somasundaram K (2005). Role of p53 status in chemosensitivity determination of cancer cells against histone deacetylase inhibitor sodium butyrate. *International Journal of Cancer*.

[54] Bukreeva EI, Aksenov ND, Bardin AA, Pospelov VA, Pospelova TV (2009). Effect of histone deacetylase inhibitor sodium butyrate (NaB) on transformants E1A+cHa-*ras* expressing wild type p53 with suppressed transactivation function. *Tsitologiia*.

[55] Matheu A, Klatt P, Serrano M (2005). Regulation of the *INK4a/ARF* locus by histone deacetylase inhibitors. *Journal of Biological Chemistry*.

[56] Wang YF, Chen NS, Chung YP, Chang LH, Chiou YH, Chen CY (2006). Sodium butyrate induces apoptosis and cell cycle arrest in primary effusion lymphoma cells independently of oxidative stress and p21^cip1/waf1^ induction. *Molecular and Cellular Biochemistry*.

[57] Kortlever RM, Bernards R (2006). Senescence, wound healing and cancer: the PAI-1 connection. *Cell Cycle*.

